# Phenyl­methanaminium chloro­acetate

**DOI:** 10.1107/S1600536809017802

**Published:** 2009-05-20

**Authors:** Durre Shahwar, M. Nawaz Tahir, Naeem Ahmad, Muhammad Akmal Khan, Asma Yasmeen

**Affiliations:** a Department of Chemistry, Government College University, Lahore, Pakistan; b Department of Physics, University of Sargodha, Sargodha, Pakistan

## Abstract

In the title compound, C_7_H_10_N^+^·C_2_H_2_ClO_2_^−^, the planar chloracetate ion [with a maximum deviation of 0.025 (3) Å] is oriented at a dihedral angle of 31.07 (4)° with respect to the planar [maximum deviation of 0.022 (3) Å] phenyl­methanaminium cation. In the crystal structure, inter­mol­ecular N—H⋯O hydrogen bonds link the mol­ecules into a network. A weak C—H⋯π inter­action is also present.

## Related literature

For related structures, see: Amini *et al.* (2007 ▶); Houllemare-Druot & Coquerel (1998 ▶); Rademeyer (2003 ▶). For bond-length data, see: Allen *et al.* (1987 ▶).
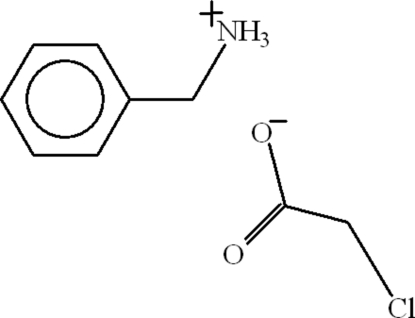


## Experimental

### 

#### Crystal data

C_7_H_10_N^+^·C_2_H_2_ClO_2_^−^
*M* *_r_* = 201.65
Orthorhombic, 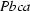

*a* = 11.1653 (9) Å
*b* = 8.0295 (5) Å
*c* = 22.3714 (18) Å
*V* = 2005.6 (3) Å^3^
*Z* = 8
Mo *K*α radiation
μ = 0.35 mm^−1^
*T* = 296 K
0.28 × 0.14 × 0.12 mm

#### Data collection

Bruker Kappa APEXII CCD area-detector diffractometer
Absorption correction: multi-scan (*SADABS*; Bruker, 2005 ▶) *T*_min_ = 0.941, *T*_max_ = 0.958
11668 measured reflections
2485 independent reflections
1592 reflections with *I* > 2σ(*I*)
*R*_int_ = 0.040

#### Refinement

*R*[*F* ^2^ > 2σ(*F* ^2^)] = 0.043
*wR*(*F* ^2^) = 0.121
*S* = 1.03
2485 reflections
128 parameters
H atoms treated by a mixture of independent and constrained refinement
Δρ_max_ = 0.21 e Å^−3^
Δρ_min_ = −0.20 e Å^−3^

### 

Data collection: *APEX2* (Bruker, 2007 ▶); cell refinement: *SAINT* (Bruker, 2007 ▶); data reduction: *SAINT*; program(s) used to solve structure: *SHELXS97* (Sheldrick, 2008 ▶); program(s) used to refine structure: *SHELXL97* (Sheldrick, 2008 ▶); molecular graphics: *ORTEP-3 for Windows* (Farrugia, 1997 ▶) and *PLATON* (Spek, 2009 ▶); software used to prepare material for publication: *WinGX* (Farrugia, 1999 ▶) and *PLATON*.

## Supplementary Material

Crystal structure: contains datablocks global, I. DOI: 10.1107/S1600536809017802/hk2686sup1.cif

Structure factors: contains datablocks I. DOI: 10.1107/S1600536809017802/hk2686Isup2.hkl

Additional supplementary materials:  crystallographic information; 3D view; checkCIF report

## Figures and Tables

**Table 1:** Hydrogen-bond geometry (Å, °)

*D*—H⋯*A*	*D*—H	H⋯*A*	*D*⋯*A*	*D*—H⋯*A*
N1—H1*A*⋯O1^i^	0.894 (16)	1.919 (17)	2.779 (2)	160.9 (16)
N1—H1*B*⋯O1^ii^	0.869 (19)	2.026 (19)	2.798 (2)	147.5 (17)
N1—H1*C*⋯O2^iii^	0.94 (2)	1.80 (2)	2.740 (2)	175.5 (16)
C5—H5⋯*Cg*1^iv^	0.93	2.93	3.777	152

Symmetry codes: (i) 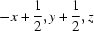; (ii) 
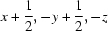
; (iii) 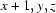; (iv) 
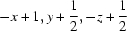
. *Cg*1 is the centroid of the benzene ring.

**Fig. 1.**
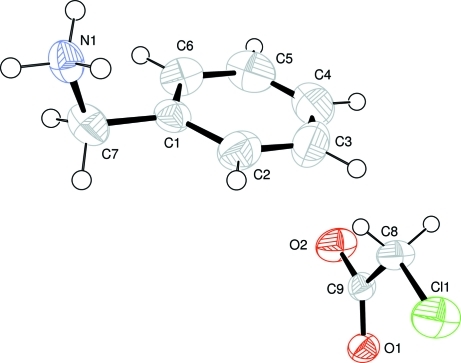
The molecular structure of the title molecule, with the atom-numbering scheme. Displacement ellipsoids are drawn at the 50% probability level.

**Fig. 2.**
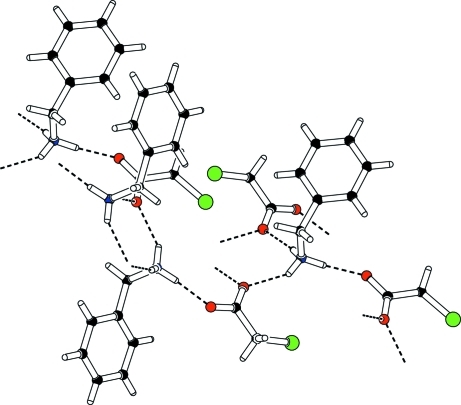
A partial packing diagram of the title compound. Hydrogen bonds are shown as dashed lines.

## supplementary crystallographic information

### Comment

Organic ammonium salts have many applications such as phase transfer catalysis, photo base-generators *etc*. We have prepared a scheme for synthesizing various ammonium salts, which will differ due to the moiety attached to NH_3_ group and due to the anion. We reported herein the crystal structure of the title compound, (I), in this regard. The crystal structures of benzylammonium nitrate, (II) (Rademeyer, 2003), bis(benzylammonium) sulfate, (III) (Amini *et al.*, 2007) and (±)-α-methylbenzylammonium chloroacetate, (IV) (Houllemare-Druot & Coquerel, 1998) have been reported.

The asymmetric unit of the title compound contains one cation and one anion (Fig. 1 ), in which the bond lengths (Allen *et al.*,  1987) and angles are within normal ranges. The planar chloracetate ion [with a maximum deviation of 0.025 (3) Å for atom C8] is oriented with respect to the planar phenylmethane moiety [with a maximum deviation of -0.022 (3) Å for atom C7] at a dihedral angle of 31.07 (4)°, and atom N1 is 1.3132 (24) Å away from the plane of the phenylmethane moiety.

In the crystal structure, strong intermolecular N-H···O hydrogen bonds (Table 1) link the molecules into a network (Fig. 2), in which they may be effective in the stabilization of the structure. There also exists a weak C—H···π interaction (Table 1).

### Experimental

For the preparation of the title compound, benzylamine (1.09 ml, 0.01 mol) was added dropwise to a solution of chloroacetic acid (0.945 g, 0.01 mol) in dichloromethane (20 ml), and stirred for 30 min. The product precipitated, filtered out and washed with n-hexane. Crystals suitable for X-ray analysis were obtained from a mixture of n-hexane/chloroform (1:1).

### Refinement

H atoms (for NH_3_) were located in difference Fourier synthesis and refined isotropically. The remaining H atoms were positioned geometrically with C-H = 0.93 and 0.97 Å, for aromatic and methylene H atoms, respectively, and constrained to ride on their parent atoms, with U_iso_(H) = 1.2U_eq_(C).

### Crystal data

**Table:**

C_7_H_10_N^+^·C_2_H_2_ClO_2_^−^	*F*(000) = 848
*M**_r_* = 201.65	*D*_x_ = 1.336 Mg m^−^^3^
Orthorhombic, *P**b**c**a*	Mo *K*α radiation, λ = 0.71073 Å
Hall symbol: -P 2ac 2ab	Cell parameters from 2485 reflections
*a* = 11.1653 (9) Å	θ = 2.6–28.3°
*b* = 8.0295 (5) Å	µ = 0.35 mm^−^^1^
*c* = 22.3714 (18) Å	*T* = 296 K
*V* = 2005.6 (3) Å^3^	Needle, colorless
*Z* = 8	0.28 × 0.14 × 0.12 mm

### Data collection

**Table:**

Bruker Kappa APEXII CCD area-detector diffractometer	2485 independent reflections
Radiation source: fine-focus sealed tube	1592 reflections with *I* > 2σ(*I*)
graphite	*R*_int_ = 0.040
Detector resolution: 7.40 pixels mm^-1^	θ_max_ = 28.3°, θ_min_ = 2.6°
ω scans	*h* = −12→14
Absorption correction: multi-scan (*SADABS*; Bruker, 2005)	*k* = −7→10
*T*_min_ = 0.941, *T*_max_ = 0.958	*l* = −29→29
11668 measured reflections	

### Refinement

**Table:**

Refinement on *F*^2^	Secondary atom site location: difference Fourier map
Least-squares matrix: full	Hydrogen site location: inferred from neighbouring sites
*R*[*F*^2^ > 2σ(*F*^2^)] = 0.043	H atoms treated by a mixture of independent and constrained refinement
*wR*(*F*^2^) = 0.121	*w* = 1/[σ^2^(*F*_o_^2^) + (0.0569*P*)^2^ + 0.2834*P*] where *P* = (*F*_o_^2^ + 2*F*_c_^2^)/3
*S* = 1.03	(Δ/σ)_max_ < 0.001
2485 reflections	Δρ_max_ = 0.21 e Å^−^^3^
128 parameters	Δρ_min_ = −0.20 e Å^−^^3^
0 restraints	Extinction correction: *SHELXL97* (Sheldrick, 2008), Fc^*^=kFc[1+0.001xFc^2^λ^3^/sin(2θ)]^-1/4^
Primary atom site location: structure-invariant direct methods	Extinction coefficient: 0.0052 (11)

### Special details

**Table:**

Geometry. Bond distances, angles *etc*. have been calculated using the rounded fractional coordinates. All su's are estimated from the variances of the (full) variance-covariance matrix. The cell e.s.d.'s are taken into account in the estimation of distances, angles and torsion angles
Refinement. Refinement of *F*^2^ against ALL reflections. The weighted *R*-factor *wR* and goodness of fit *S* are based on *F*^2^, conventional *R*-factors *R* are based on *F*, with *F* set to zero for negative *F*^2^. The threshold expression of *F*^2^ > σ(*F*^2^) is used only for calculating *R*-factors(gt) *etc*. and is not relevant to the choice of reflections for refinement. *R*-factors based on *F*^2^ are statistically about twice as large as those based on *F*, and *R*- factors based on ALL data will be even larger.

### Fractional atomic coordinates and isotropic or equivalent isotropic displacement parameters (Å^2^)

**Table:**

	*x*	*y*	*z*	*U*_iso_*/*U*_eq_	
Cl1	0.22460 (4)	0.33171 (7)	0.07550 (3)	0.0665 (2)	
O1	−0.01235 (10)	0.22583 (15)	0.03185 (6)	0.0435 (4)	
O2	−0.11363 (11)	0.44211 (18)	0.06754 (7)	0.0586 (5)	
N1	0.64467 (14)	0.4356 (2)	0.04653 (7)	0.0368 (5)	
C1	0.59953 (15)	0.4607 (2)	0.15510 (8)	0.0376 (5)	
C2	0.69962 (18)	0.4421 (3)	0.19030 (10)	0.0519 (7)	
C3	0.7096 (2)	0.5291 (3)	0.24323 (11)	0.0684 (9)	
C4	0.6222 (3)	0.6365 (3)	0.26080 (10)	0.0693 (9)	
C5	0.5239 (2)	0.6594 (3)	0.22598 (11)	0.0634 (8)	
C6	0.51145 (17)	0.5705 (2)	0.17352 (9)	0.0492 (6)	
C7	0.58321 (18)	0.3615 (2)	0.09867 (9)	0.0495 (7)	
C8	0.08928 (14)	0.4447 (2)	0.08390 (9)	0.0436 (6)	
C9	−0.02110 (14)	0.3609 (2)	0.05846 (7)	0.0333 (5)	
H1A	0.6185 (16)	0.540 (2)	0.0418 (8)	0.0442*	
H1B	0.6233 (17)	0.376 (2)	0.0159 (9)	0.0442*	
H1C	0.7280 (18)	0.436 (2)	0.0517 (8)	0.0442*	
H2	0.76068	0.37060	0.17840	0.0623*	
H3	0.77687	0.51420	0.26716	0.0821*	
H4	0.62979	0.69415	0.29664	0.0832*	
H5	0.46510	0.73487	0.23747	0.0761*	
H6	0.44308	0.58455	0.15031	0.0590*	
H7A	0.49832	0.35274	0.08999	0.0593*	
H7B	0.61368	0.24974	0.10502	0.0593*	
H8A	0.07616	0.46464	0.12616	0.0523*	
H8B	0.09865	0.55221	0.06471	0.0523*	

### Atomic displacement parameters (Å^2^)

**Table:**

	*U*^11^	*U*^22^	*U*^33^	*U*^12^	*U*^13^	*U*^23^
Cl1	0.0303 (3)	0.0642 (4)	0.1049 (5)	0.0065 (2)	−0.0105 (3)	−0.0122 (3)
O1	0.0383 (7)	0.0417 (7)	0.0504 (7)	−0.0084 (5)	0.0054 (5)	−0.0141 (6)
O2	0.0301 (7)	0.0623 (10)	0.0834 (11)	0.0074 (6)	−0.0015 (6)	−0.0179 (8)
N1	0.0312 (7)	0.0368 (9)	0.0424 (9)	0.0039 (7)	−0.0020 (7)	−0.0106 (7)
C1	0.0378 (9)	0.0337 (9)	0.0412 (10)	−0.0065 (8)	0.0030 (8)	0.0045 (8)
C2	0.0450 (11)	0.0533 (13)	0.0573 (13)	0.0005 (9)	−0.0035 (9)	0.0111 (10)
C3	0.0693 (15)	0.0801 (17)	0.0558 (14)	−0.0243 (14)	−0.0217 (12)	0.0170 (13)
C4	0.105 (2)	0.0624 (16)	0.0404 (12)	−0.0273 (15)	0.0086 (13)	−0.0014 (10)
C5	0.0784 (16)	0.0544 (14)	0.0575 (13)	0.0027 (12)	0.0264 (13)	−0.0040 (11)
C6	0.0416 (10)	0.0540 (12)	0.0519 (11)	0.0062 (9)	0.0067 (9)	0.0063 (9)
C7	0.0511 (11)	0.0411 (11)	0.0562 (12)	−0.0094 (9)	0.0043 (10)	−0.0049 (9)
C8	0.0320 (8)	0.0410 (11)	0.0577 (11)	0.0024 (8)	−0.0047 (8)	−0.0138 (9)
C9	0.0293 (8)	0.0355 (10)	0.0350 (9)	−0.0021 (7)	0.0009 (7)	−0.0020 (8)

### Geometric parameters (Å, °)

**Table:**

Cl1—C8	1.7723 (17)	C4—C5	1.358 (4)
O1—C9	1.241 (2)	C5—C6	1.381 (3)
O2—C9	1.239 (2)	C2—H2	0.9300
N1—C7	1.478 (3)	C3—H3	0.9300
N1—H1A	0.894 (16)	C4—H4	0.9300
N1—H1B	0.869 (19)	C5—H5	0.9300
N1—H1C	0.94 (2)	C6—H6	0.9300
C1—C6	1.384 (2)	C7—H7A	0.9700
C1—C2	1.375 (3)	C7—H7B	0.9700
C1—C7	1.504 (3)	C8—C9	1.515 (2)
C2—C3	1.379 (3)	C8—H8A	0.9700
C3—C4	1.360 (4)	C8—H8B	0.9700
			
Cl1···O1	2.9455 (13)	C5···H3^xii^	3.0000
Cl1···H7A	3.0800	C6···H3^xii^	2.9700
Cl1···H1B^i^	2.871 (19)	C9···H1C^iii^	2.87 (2)
Cl1···H8B^ii^	3.0000	C9···H1B^i^	2.998 (18)
O1···Cl1	2.9455 (13)	C9···H1A^ii^	2.822 (16)
O1···N1^i^	2.798 (2)	C9···H8B^ix^	2.9700
O1···C7^i^	3.187 (2)	H1A···O1^iv^	1.919 (17)
O1···C7^ii^	3.379 (2)	H1A···C9^iv^	2.822 (16)
O1···N1^ii^	2.779 (2)	H1B···Cl1^vi^	2.871 (19)
O1···C6^ii^	3.406 (2)	H1B···O1^vi^	2.026 (19)
O2···N1^iii^	2.740 (2)	H1B···C9^vi^	2.998 (18)
O1···H7A^i^	2.8000	H1C···O2^v^	1.80 (2)
O1···H1A^ii^	1.919 (17)	H1C···C9^v^	2.87 (2)
O1···H1B^i^	2.026 (19)	H2···H7B	2.5200
O2···H1C^iii^	1.80 (2)	H2···O2^v^	2.9100
O2···H2^iii^	2.9100	H2···C4^vii^	2.9400
O2···H7B^iv^	2.6100	H3···C6^xi^	2.9700
N1···O1^iv^	2.779 (2)	H3···C5^xi^	3.0000
N1···O2^v^	2.740 (2)	H4···H8A^xi^	2.6000
N1···O1^vi^	2.798 (2)	H5···C2^xiii^	2.9600
C2···C4^vii^	3.531 (4)	H5···C3^xiii^	3.0900
C4···C2^viii^	3.531 (4)	H5···C1^xiii^	3.1000
C6···O1^iv^	3.406 (2)	H6···H7A	2.3800
C6···C9^iv^	3.475 (2)	H7A···Cl1	3.0800
C7···O1^iv^	3.379 (2)	H7A···H6	2.3800
C7···O1^vi^	3.187 (2)	H7A···O1^vi^	2.8000
C9···C9^ix^	3.472 (2)	H7B···H2	2.5200
C9···C6^ii^	3.475 (2)	H7B···O2^ii^	2.6100
C1···H5^x^	3.1000	H8A···C4^xii^	2.9300
C2···H5^x^	2.9600	H8A···H4^xii^	2.6000
C3···H5^x^	3.0900	H8B···C9^ix^	2.9700
C4···H2^viii^	2.9400	H8B···Cl1^iv^	3.0000
C4···H8A^xi^	2.9300		
			
H1B—N1—H1C	111.8 (16)	C5—C4—H4	120.00
C7—N1—H1C	111.4 (11)	C4—C5—H5	120.00
C7—N1—H1A	108.6 (12)	C6—C5—H5	120.00
C7—N1—H1B	105.8 (12)	C1—C6—H6	120.00
H1A—N1—H1B	109.5 (16)	C5—C6—H6	120.00
H1A—N1—H1C	109.6 (15)	N1—C7—H7A	109.00
C2—C1—C7	121.44 (17)	N1—C7—H7B	109.00
C2—C1—C6	118.44 (18)	C1—C7—H7A	109.00
C6—C1—C7	120.10 (16)	C1—C7—H7B	109.00
C1—C2—C3	120.2 (2)	H7A—C7—H7B	108.00
C2—C3—C4	120.7 (2)	Cl1—C8—C9	115.23 (12)
C3—C4—C5	120.0 (2)	O1—C9—O2	127.19 (15)
C4—C5—C6	119.9 (2)	O1—C9—C8	120.28 (14)
C1—C6—C5	120.74 (18)	O2—C9—C8	112.53 (14)
N1—C7—C1	113.14 (14)	Cl1—C8—H8A	108.00
C1—C2—H2	120.00	Cl1—C8—H8B	108.00
C3—C2—H2	120.00	C9—C8—H8A	108.00
C2—C3—H3	120.00	C9—C8—H8B	108.00
C4—C3—H3	120.00	H8A—C8—H8B	108.00
C3—C4—H4	120.00		
			
C6—C1—C2—C3	1.3 (3)	C1—C2—C3—C4	−1.3 (4)
C7—C1—C2—C3	−177.00 (19)	C2—C3—C4—C5	−0.2 (4)
C2—C1—C6—C5	0.1 (3)	C3—C4—C5—C6	1.6 (4)
C7—C1—C6—C5	178.42 (18)	C4—C5—C6—C1	−1.6 (3)
C2—C1—C7—N1	−83.7 (2)	Cl1—C8—C9—O1	−2.8 (2)
C6—C1—C7—N1	98.1 (2)	Cl1—C8—C9—O2	177.43 (13)

Symmetry codes: (i) *x*−1/2, −*y*+1/2, −*z*; (ii) −*x*+1/2, *y*−1/2, *z*; (iii) *x*−1, *y*, *z*; (iv) −*x*+1/2, *y*+1/2, *z*; (v) *x*+1, *y*, *z*; (vi) *x*+1/2, −*y*+1/2, −*z*; (vii) −*x*+3/2, *y*−1/2, *z*; (viii) −*x*+3/2, *y*+1/2, *z*; (ix) −*x*, −*y*+1, −*z*; (x) −*x*+1, *y*−1/2, −*z*+1/2; (xi) *x*+1/2, *y*, −*z*+1/2; (xii) *x*−1/2, *y*, −*z*+1/2; (xiii) −*x*+1, *y*+1/2, −*z*+1/2.

### Hydrogen-bond geometry (Å, °)

**Table:**

*D*—H···*A*	*D*—H	H···*A*	*D*···*A*	*D*—H···*A*
N1—H1A···O1^iv^	0.894 (16)	1.919 (17)	2.779 (2)	160.9 (16)
N1—H1B···O1^vi^	0.869 (19)	2.026 (19)	2.798 (2)	147.5 (17)
N1—H1C···O2^v^	0.94 (2)	1.80 (2)	2.740 (2)	175.5 (16)
C5—H5···Cg1^xiii^	0.93	2.93	3.777	152.00

Symmetry codes: (iv) −*x*+1/2, *y*+1/2, *z*; (vi) *x*+1/2, −*y*+1/2, −*z*; (v) *x*+1, *y*, *z*; (xiii) −*x*+1, *y*+1/2, −*z*+1/2.

## References

[bb1] Allen, F. H., Kennard, O., Watson, D. G., Brammer, L., Orpen, A. G. & Taylor, R. (1987). *J. Chem. Soc. Perkin Trans. 2*, pp. S1–19.

[bb2] Amini, M. M., Nasiri, S. & Ng, S. W. (2007). *Acta Cryst.* E**63**, o1361–o1362.

[bb3] Bruker (2005). *SADABS* Bruker AXS Inc., Madison, Wisconsin, USA.

[bb4] Bruker (2007). *APEX2* and *SAINT* Bruker AXS Inc., Madison, Wisconsin, USA.

[bb5] Farrugia, L. J. (1997). *J. Appl. Cryst.* **30**, 565.

[bb6] Farrugia, L. J. (1999). *J. Appl. Cryst.* **32**, 837–838.

[bb7] Houllemare-Druot, S. & Coquerel, G. (1998). *J. Chem. Soc. Perkin Trans. 2*, pp. 2211–2220.

[bb8] Rademeyer, M. (2003). *Acta Cryst.* E**59**, o1860–o1861.

[bb9] Sheldrick, G. M. (2008). *Acta Cryst.* A**64**, 112–122. 10.1107/S0108767307043930 18156677

[bb10] Spek, A. L. (2009). *Acta Cryst.* D**65**, 148–155. 10.1107/S090744490804362X PMC2631630 19171970

